# Novel Synthesis and Antitumor Evaluation of Polyfunctionally Substituted Heterocyclic Compounds Derived from 2-Cyano-*N*-(3-cyano-4,5,6,7-tetrahydrobenzo[*b*]thiophen-2-yl)-acetamide

**DOI:** 10.3390/molecules16010052

**Published:** 2010-12-27

**Authors:** Hoda Z. Shams, Rafat M. Mohareb, Maher H. Helal, Amira E. Mahmoud

**Affiliations:** 1 Department of Chemistry, Faculty of Science, Helwan University, Ain Helwan, Cairo, Egypt; 2 Department of Organic Chemistry, Faculty of Pharmacy, October University for Modern Sciences and Arts, October City, Egypt; 3 Department of Chemistry, Faculty of Science, Cairo University, Giza, Egypt

**Keywords:** 4,5,6,7-tetrahydrobenzo[*b*]thiophene, thiazole, pyrazole, pyridine, antitumor

## Abstract

The reaction of 2-amino-3-cyano-4,5,6,7-tetrahydrobenzo[*b*]thiophene with ethyl cyanoacetate gave 2-cyano-*N*-(3-cyano-4,5,6,7-tetrahydrobenzo[*b*]thiophen-2-yl)-acetamide. The latter was used to synthesize different heterocyclic derivatives comprising thiophene, thiazole, pyrazole, pyridine, pyrimidine, and coumarin rings. The mechanistic and synthetic pathways depended on regioselective attack and/or cyclization by the cyanoacetamido moiety in the key precursor on various chemical reagents. The competition of the reaction pathways including dipolar cyclization, dinucleophilic-bielectrophilic attack, β-attack, Gewald-type attack, and condensation reactions led to the diversity of the synthesized products. The antitumor activities of the synthesized products were studied and evaluated. Most of the compounds revealed high inhibitory effects when screened *in vitro* for their antiproliferative activity. Three human cancer cell lines, namely, breast adenocarcinoma (MCF-7), non-small cell lung cancer (NCI-H460) and CNS cancer (SF-268) were used in the screening tests. The simplicity of the synthetic procedures which mainly involved one-pot reactions under mild reaction conditions, the convenience of yield production and the diversity of the reactive sites in the produced systems play a valuable role for further heterocyclic transformations and further biological investigations.

## 1. Introduction

Benzothiophene systems and their substituted derivatives have attracted a great deal of interest over the years. Their aromatic character contributes to their reactivity, stability and chemical and electronic properties. A vast number of heterocyclic derivatives observed in natural products have beenreported [1,2]. On the other side, they find increasing application as superconductors [3,4], optoelectronics [5,6], light emission diodes LEDs, and non-linear optical (NLO) chromophores [7,8]. Besides, their pharmacological profiles as antimicrobial [9,10], antifungal [11], antiinflammatory [12], antiproliferative [13,14,15] and antioxidant [16] agents have led to an enduring interest in the development of various methods for their synthesis. The most efficient protocols for carrying out the synthesis of such thiophene derivatives are the Gewald method [17,18], intramolecular cyclization via both nucleophilic displacement [14,19] and thio-Claisen rearrangement [20], and dehydrophoto- cyclization [21,22]. Of particular interest are methods that utilize new classes of precursors. Among the many synthetic methods available are C-C bond formation using Montmorillonite K-10 as Friedel Crafts catalyst [23]. C-C and C-N bond formation via transition metal catalyzed processes involving palladium-mediated cross-coupling cyclizations (e.g., Suzuki, Sonogashira and Buchwald-Hartwig cross coupling), also feature heavily [24,25,26,27,28]. Within the scope of these diverse synthetic methods and the utility of thiophene-based systems and in continuation to our interest in the design of bioactive heterocycles [29,30,31], we focused our efforts to developing novel highly substituted and polyfunctional heterocyclic compounds based on the key precursor 2-cyano-*N*-(3-cyano-4,5,6,7-tetrahydrobenzo[*b*]-thiophen-2-yl)-acetamide (**2**) and evaluating their antitumor activities.

## 2. Results and Discussion

### 2.1. Chemistry

**Scheme 1:**
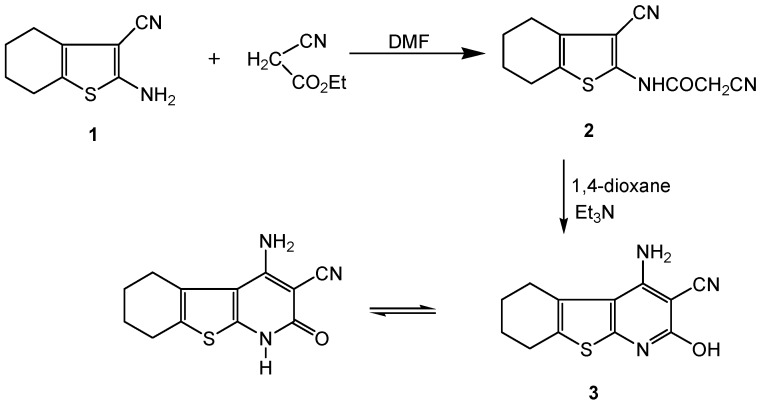
Synthesis of the precursor **2** and its cyclized product **3**.

**Scheme 2:**
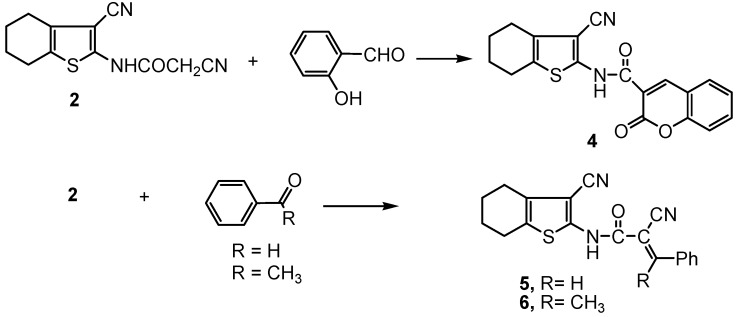
Synthesis of the coumarin derivative **4** and benzal derivatives **5** and **6**.

**Scheme 3:**
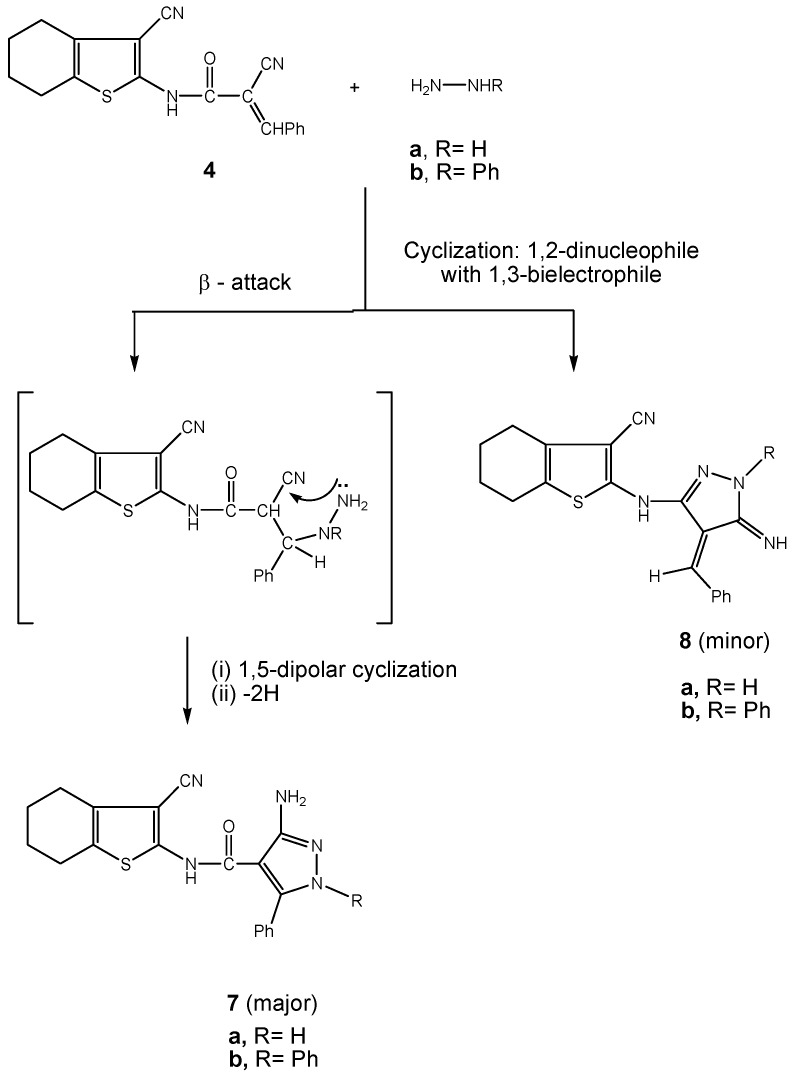
Synthesis of pyrazole systems **7a,b**.

**Scheme 4:**
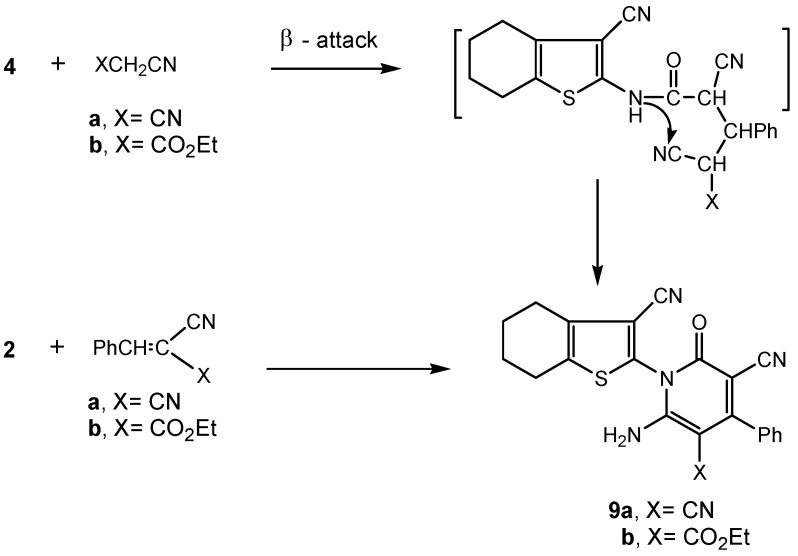
Synthesis of pyridone derivatives **9a,b**.

**Scheme 5:**
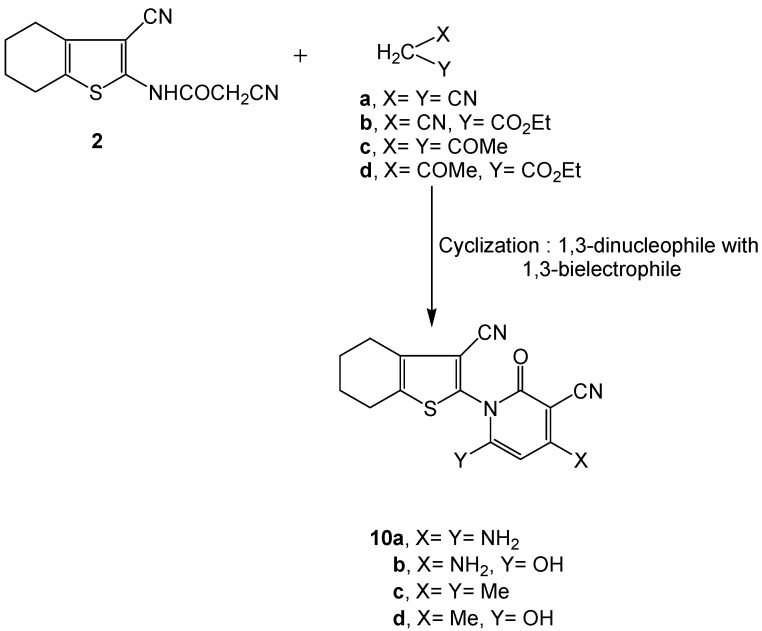
Synthesis of 2-pyridone derivatives **10a-d**.

**Scheme 6:**
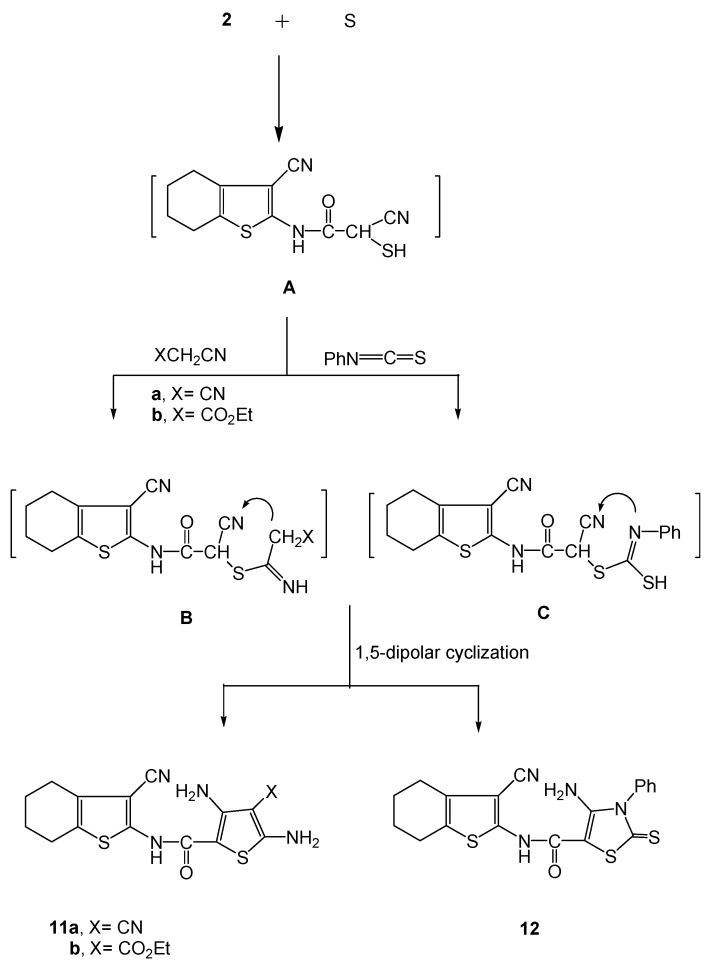
Synthesis of the highly functionalized thiophenes **11a,b** and thiazole **12**.

**Scheme 7:**
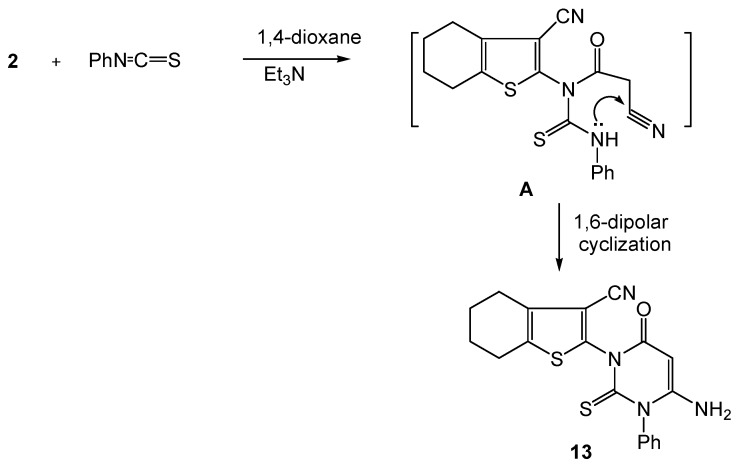
Synthesis of 2-pyrimidone-6-thione **13**.

**Scheme 8:**
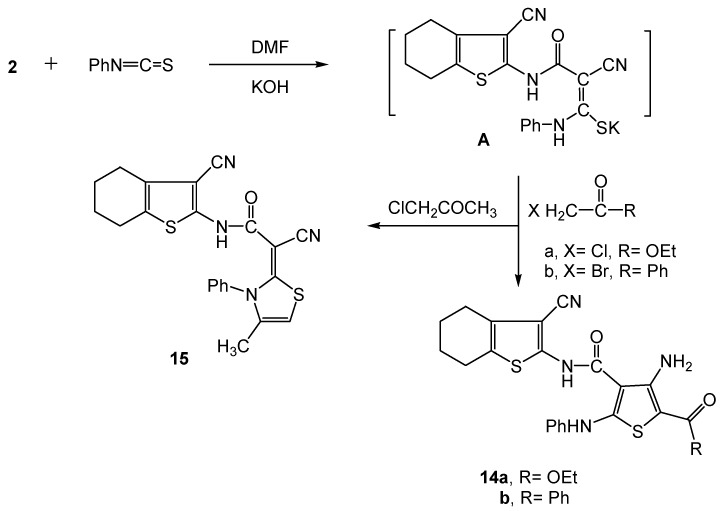
Synthesis of thiophene derivatives **14a,b** and the thiazole **15**.

**Scheme 9:**
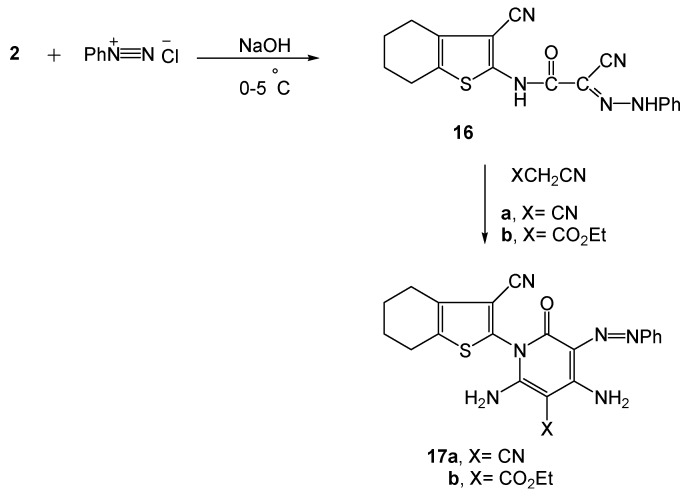
Synthesis of 2-pyridone-3-phenylazo derivatives **17a,b**.

The synthetic strategies adopted to obtain the newly synthesized compounds **3-17** depended on the regioselective attack on the cyanoacetamido moiety of the key precursor **2** by different reagents, which, in one or two steps added a highly functionalized substituent or heterocyclic ring to the molecule. The mechanistic pathways for our protocol are outlined in Scheme 1, Scheme 2, Scheme 3, Scheme 4, Scheme 5, Scheme 6, Scheme 7, Scheme 8 and Scheme 9. CHNS microanalytical data, IR, ^1^H-NMR and MS spectral data are indicated in the Experimental section.

The reaction of 2-amino-3-cyano-4,5,6,7-tetrahydrobenzo[*b*]thiophene (**1**) [32] with ethyl cyano-acetate gave 2-cyano-*N*-(3-cyano-4,5,6,7-tetrahydrobenzo[*b*]thiophen-2-yl)-acetamide **2** ( Scheme 1). The IR spectrum of **2** revealed two CN stretching bands at 2,262 and 2,196 cm^−1^, and a characteristic C=O stretching at 1,696 cm^−1^. Moreover, the ^1^H-NMR spectrum exhibited a multiplet due to four CH_2_ groups at δ 1.70-2.60, a singlet at δ 4.11 for the acetamido CH_2_ and a singlet at δ 6.94 ppm for the amidic NH. GC-MS analysis of compound **2** exhibited a [M^+^] ion (*m/z* 245), confirming the molecular weight of this compound. The peak at *m/z* 178 indicated the fragmentation of [COCH_2_CN]^+^ (*m/z* 68) from [M^+^ + 1]. The base peak observed at *m/z* 150 represented the fragmentation of [CNHCOCH_2_CN]^+^ (*m/z* 95) from the [M^+^] ion. Compound **2** underwent ready cyclization when heated in 1,4-dioxane containing triethylamine to give the tetrahydrobenzo[4,5]thieno[2,3-*b*]pyridine derivative **3** (Scheme 1).

The absence of one of the two CN functions (IR) and of δ singlet CH_2_ (^1^H NMR) observed in compound **2** as well as the appearance of a NH_2 _singlet at δ 3.61 ppm (^1^H-NMR) confirmed the fused structure **3**. It is noteworthy that the IR spectrum of compound **3** showed a C=O stretching band at 1,621 cm^−1 ^and its ^1^H-NMR showed a D_2_O exchangeable singlet at δ 6.97 ppm corresponding to the NH group, confirming that **3** exists in both keto and enol forms. Compound **3** revealed a [M^+^] (*m/z* 245) which is also the base peak.

The reactivity of compound **2** towards various chemical reagents was investigated with the aim to producing thiophene systems with potential biological activities. Thus, the reaction of **2** with salicylaldehyde gave the coumarin derivative **4**. On the other hand, the reaction of **2** with either benzaldehyde or acetophenone gave the benzylidene derivatives **5** and **6**, respectively (Scheme 2).

All collected data for compounds **4-6** were consistent with the proposed structures. Thus, the observation of δ-^1^H benzylidine C=CH at 8.56 ppm in the ^1^H-NMR spectrum of **5** and a coumarin C4-H proton at δ 6.94 ppm in the ^1^H-NMR spectrum of **4**, as well as the appearance of a high frequency C=O stretching at 1,715 cm^−1^ cited for the coumarin oxo function besides the absence of the cyanoacetamido CN observed in the starting material **2**, in the IR spectrum of **4** proved the expected structures. Compounds **5** and **4** revealed molecular ion peaks [M^+^] at *m/z* 333 and [M^+^ − 1] (*m/z* 349), corresponding to the molecular formulae C_19_H_15_N_3_OS and C_19_H_14_N_2_O_3_S, respectively. A base peak at *m/z* 266 due to splitting of the fragment ion [C(=O)-C-CN]^+^ (*m/z* 66) from the [M^+^ − 1] ion of **5**, and a base peak at *m/z* 172 which resulted from the fragmentation of [3-CN-4,5,6,7-tetrahydrobenzo[*b*]thiophene-2-NH]^+^ (*m/z* 177) from the [M^+^ − 1] ion of **4** were observed in their respective mass spectra. 

When compound **2** was reacted with acetophenone in the presence of ammonium acetate in an oil bath at 140 °C the Knoevenagel condensation product **6** was obtained (Scheme 2). The structure of compound **6** was based on analytical and spectral data (see Experimental section). Moreover, the GC-MS spectrum of compound **6** revealed a molecular ion peak [M^+^ − 1] at *m/z* 346, a base peak at *m/z* 150, resulting from the fragmentation of [CNHC(=O)C(CN)=C(CH_3_Ph)]^+^ (*m/z* 197) from the [M^+^] ion, and a fragment ion peak at *m/z* 178 due to the fragmentation of [C(=O)C(CN)=C(CH_3_Ph)]^+^ (*m/z* 170) from the [M^+^ + 1] ion peak.

When compound **4** reacted with either hydrazine hydrate or phenyl hydrazine, the respective pyrazole systems **7a,b** were obtained as the major products (Scheme 3). The reaction involves β-attack on the C(=O)C=C moiety in **4** with subsequent 1,5-intramolecular dipolar cyclization and concomitant aromatization.

Minor products **8a,b** were also obtained through the first condensation with the amide C=O followed by cyclization. Microanalysis and spectral data of **7a,b** were fully consistent with the proposed structures. The mass spectrum of the pyrazole system **7b** exhibited a molecular ion peak [M^+^] (*m/z* 439) corresponding to molecular formula C_25_H_21_N_5_OS.

On the other hand, treatment of the benzylidene derivative **4** with methylene carbonitrile reagents (XCH_2_CN; X = CN, X = CO_2_Et) afforded the respective pyridone derivatives **9a,b** (Scheme 4). The reaction took place via β-attack on the benzylidene moiety in **4** followed by 1,6-intramolecular dipolar cyclization with concomitant aromatization. The IR spectrum of **9a** revealed the presence of three CN stretching bands at υ 2,253, 2,223 and 2,209 cm^−1^. Moreover, the ^1^H-NMR spectra of **9a** and **9b** showed the presence of one singlet for each at δ 3.61 and δ 3.41 ppm, respectively, due to the presence of the NH_2_ group. Compound **9b** showed a triplet at δ 1.21 for the ester CH_3_ group and a quartet at δ 4.30 corresponding to the ester CH_2_ group. Moreover, in the mass spectrum of 9a the existing [M^+^] ion (*m/z* 397) corresponding to the formula C_22_H_15_N_5_OS and representing the base peak was observed, whereas the mass spectrum of **9b** exhibited a molecular ion peak [M^+^] at *m/z* = 444 confirming its molecular formula C_24_H_20_N_4_O_3_S.

Further confirmation of the reaction products **9a,b** was achieved through an alternative synthetic route involving treatment of the starting compound **2** with benzylidene carbonitrile reagents (PhCH=C(CN)X; X = CN; X = CO_2_Et) to afford the same pyridone derivatives **9a,b** (verified by IR fingerprint, m.p. and mixed m.p.) with better yields (80%, 86%) than in their formation by the reaction of compound **4** and either malononitrile (75% yield) or ethyl cyanoacetate (73% yield) (Scheme 4).

By subjecting the starting material **2** to reaction with active methylene reagents (X CH_2_Y; X = Y = CN; X = CN, Y = CO_2_Et; X = Y = COCH_3_ or X = COCH_3_, Y = CO_2_Et) the respective 2-pyridone derivatives **10a-d** were obtained (Scheme 5).

All data for compounds **10a-d** were consistent with the proposed structures. Thus, the absence of the δ-^1^H CH_2_ cited for the acetamido methylene protons observed with **2** at δ 4.11 ppm and the appearance of the pyridine C5-H protons at δ 7.89, 6.92, 6.51 and 6.88 ppm in the respective ^1^H-NMR spectra of **10a-d** confirmed the proposed structures. Moreover, the CH_3_ proton singlets were recorded at δ 2.11 and 2.36 ppm with compound **10c** and at δ 2.55 ppm with **10d**, whereas the δ-^1^H signals for OH were integrated at 11.89 and 12.10 ppm in the respective ^1^H-NMR spectra of compounds **10b,d**. The mass spectra of **10a,b** exhibited a common peak at *m/z* 311 which corresponded to their [M^+^] and [M^+^ − 1] ions, respectively. Another common peak was also observed at *m/z* 296 due to splitting of NH_2_ from their respective [M^+^ + 1] and [M^+^] molecular ions. The peak observed for **10a** at *m/z* 282 is due to the fragmentation of two NH_2_ from [M^+^ + 3], while that observed for **10b** at *m/z* 285 corresponded to the loss of CN group from its [M^+^ − 1]. The mass spectrum of **10b** exhibited two fragment ion peaks at *m/z* 219 and 164 due to fragmentation of [C(=O)-C(CN)=C-(NH_2_)]^+^ from [M^+^ + 1] and of functionalized pyridone (C_6_H_5_N_3_O_2_) from [M^+^ + 3]. The mass spectrum of **10c** displayed a [M^+^] base peak at *m/z* 309 corresponding to the molecular formula C_17_H_15_N_3_OS. Compound **10c** revealed two main fragment ions at *m/z* 162 and *m/z* 147 due to splitting of 3-CN-4,5,6,7-tetrahydrobenzo[*b*]-thiophene [C_9_H_8_NS]^+^ and the functionalized pyridone C_8_H_7_N_2_O fragments, respectively.

The peak observed at *m/z* 131 is due to the fragmentation of 2-amino-3-cyano-4,5,6,7-tetrahydrobenzo[*b*]thiophenyl cation [C_9_H_10_N_2_S]^+^ from the molecular ion peak [M^+^] of **10c** whereas that observed at *m/z* 281 resulted from fragmentation of CN from [M^+^ − 2]. On the other hand, compound **10d** exhibited [M^+^ + 2] at *m/z* 313 indicating a molecular formula C_16_H_13_N_3_O_2_S, and a base peak *m/z* 219 due to fragmentation of [C(=O)-C(CN)=C(CH_3_)]^+^ (*m/z* 93) from its [M^+^ + 1]. Two fragments observed at *m/z* 163 and *m/z* 150 resulted from splitting of 3-cyano-4,5,6,7-tetrahydrobenzo-[*b*]thiophenyl cation [C_9_H_9_NS]^+^ and functionalized pyridone [C_7_H_6_N_2_O_2_]^+^, respectively.

At the other extreme, when compound **2** reacted with elemental sulfur and either of the methylene carbonitrile reagents (X-CH_2_-CN or X = CN; X = CO_2_Et) it gave the thiophene derivatives **11a,b**, respectively. The reaction of compound **2** with phenyl isothiocyanate and elemental sulfur gave the thiazole-2-thione derivative **12**. Formation of **11a,b** took place through intermediate formation of A and B, while the formation of **12** occurred through intermediacy of A and C (Scheme 6).

The ^1^H-NMR spectrum of compound **11a** revealed the existence of two singlets at δ 3.34 and 3.38 ppm corresponding to two NH_2_ groups, while compound **11b** showed two singlets at δ 3.31 and 3.35 ppm corresponding to the two NH_2_ groups, a triplet at δ 1.12 for the ester CH_3_ group and a quartet at δ 3.49 for the ester CH_2_ group. GC-MS analysis of **11a,b** and **12** showed molecular ion peaks [M^+^ + 3] *m/z* 346, [M^+^ + 2] *m/z* 392 and [M^+^] *m/z* 412. The main fragmentation of **11a,b** and **12** gave the most abundant peak recorded at *m/z* 150, which corresponded to the fragment ion [C_8_H_8_NS]^+^ resulting from the fragmentation of [3,5-di-NH_2_-4-CN-thiophene-2-CONHC]^+^ (*m/z* 193), [3,5-di-NH_2_-4-C(=O)OEt-thiophene-2-CONHC]^+^ (*m/z* 240) and [4-NH_2_-3-Ph-2-thione-2,3- dihydrothiazole-5-CONHC]^+^ (*m/z* 262), respectively, from their corresponding molecular ions [M^+^]. The peak observed at *m/z* 150 represented the base peak for **11a**. A base peak observed at *m/z* 362 in the mass spectrum of **11b** indicated the fragmentation of [C-NH_2_]^+^ (*m/z* 28) from the [M^+^] ion, whereas the base peak observed at *m/z* 93 in the mass spectrum of **12** corresponded to the cleavage of Ph-NH_2_. A common peak observed at *m/z* 178 in the mass spectra of **11a,b** and **12** indicated the loss of 2-NH_2_-3-CN-4,5,6,7-tetrahydrobenzo[*b*]-thiophene.

Next, we moved to the studying of the the reaction of compound **2** with phenyl isothiocyanate in 1,4-dioxane containing triethylamine. The reaction involved a nucleophilic attack by the amidic NH function in **2** on the C=S terminal of the isocyanate reagent to produce the acyclic intermediate **A**. The latter then underwent 1,6-dipolar cyclization to afford the 6-thioxo-2-pyrimidone derivative **13** as the major product (Scheme 7).

The data obtained from the IR, ^1^H-NMR and MS spectra are in agreement with the proposed structure. Thus, the ^1^H-NMR of compound **13** exhibited singlets at δ 6.92 and 3.83 ppm due to pyrimidine C3-H and NH_2_, respectively. The mass spectrum of **13** revealed a [M^+^ + 2] ion peak at *m/z* 382, a base peak at *m/z* 313 due to fragmentation of [C(=O)-CH=C-NH_2_]^ +^ (*m/z* 69) from the [M^+^ + 2] as well as a fragment ion at *m/z* 255 due to fragmentation of [C(=S)=N-C(=O)-C=C-NH_2_]^+^ (*m/z* 126) from [M^+^ + 1].

On the other hand, we are involved in a comprehensive programme studying the reactivity of active methylene reagents towards phenyl isothiocyanate in basic dimethylformamide followed by heterocyclization with α-halocarbonyl compounds. These reactions lead to the formation of either thiophene or thiazole systems or both, depending on reaction conditions and the nature of the α-halo-carbonyl reagent [33]. Thus, subjecting the active methylene key precursor **2** to the aforementioned reaction using α-halocarbonyl reagents XCH_2_C(=O)R (X = Cl, R = OEt; X = Br, R = Ph; X = Cl, R = CH_3_) afforded the functionalized thiophene and thiazole derivatives **14a,b** and **15**, respectively (Scheme 8). The reaction took place through the intermediacy of the potassium sulphide salt **A**. The disappearance of δ-^1^H CH_2_ singlet observed in the precursor **2**, and the appearance of D_2_O exchangeable NH_2 _singlets at δ 4.37 and δ 4.80 ppm for compounds **14a** and **14b**, respectively, as well as the appearance of a δ-^1^H singlet at 6.67 ppm assigned to a thiazole C_5_-H proton in compound **15** are considered sufficient proof for the structures of **14a,b** and **15**. Moreover, The mass spectra of **14a,b** and **15** displayed [M^+^] ion peaks at *m/z* 466, 498 and 418, respectively, corresponding to their respective molecular formulae C_23_H_22_N_4_O_3_S_2_, C_27_H_22_N_4_O_2_S_2_ and C_22_H_18_N_4_OS_2_. The main fragmentation of the title compounds revealed three important common peaks: *m/z* 77 due to [phenyl]^+^, *m/z* 178 due to 2-NH_2_-3-CN-4,5,6,7-tetrahydrobenzo[*b*]thiophene, and *m/z* 150 to [C_8_H_8_NS]^+^. Such *m/z* peaks resulted from the fragmentation of [3-NH_2_-2-COOEt-5-NHPh-thiophene-4-CONH-C]^+^ (*m/z* 316), [3-NH_2_-2-COPh-5-NHPh-thiophene-4-CONH-C]^+^ (*m/z* 348) and [4-CH_3_-3-Ph-3H-thiazol-2-ylidine-C-(CN)-CONH-C]^+^ (*m/z* 268) from their corresponding [M^+^] ions. The peak observed at *m/z* 77 due to [phenyl]^+^ represented the base peak for both compounds **14a,b**.

Compound **15** displayed a base peak at *m/z* 241 due to the loss of a 2-amino-3-cyano-4,5,6,7-tetra-0hydrobenzo[*b*]thiophenyl cation (*m/z* 178) from its [M^+^ + 1] molecular ion. Other fragment ions were observed in the mass spectra of **14a,b** and **15** at their expected *m/z* values.

On the other hand, compound **2** reacted with benzenediazonium chloride to give the phenylhydrazo derivative **16**. The latter compound reacted with either malononitrile or ethyl cyanoacetate to give the 3-phenylazo-pyridone derivatives **17a** and **17b**, respectively (Scheme 9). The structural assignments of **16**, **17a,b** were based on analytical and spectral data. Thus, the ^1^H-NMR spectrum of compound **16** revealed two singlets at δ 9.14 and 10.88 ppm (D_2_O exchangeable) due to the two NH groups. Moreover, the mass spectrum of **16** showed a molecular ion peak at *m/z* 349 [M^+^], and a base peak at *m/z* 178 which corresponded to the loss of [C(=O)-C(CN)=N-NHPh]^+^ (*m/z* 172) from [M^+^ + 1]. Fragment ions at *m/z* 149 due to fragmentation of [C-(NH)-C(=O)-C(CN)=N-NHPh]^+^ (*m/z* 197) from [M^+^ − 1], and the peak at *m/z* 167 resulting from fragment ion [NH-C(=O)-C(CN)=N-NHPh]^+^ (*m/z* 187) from [M^+^ + 5].

Common features of compounds **17a,b** are their strong carbonyl absorptions around 1,680 cm^−1^ in their IR spectra, in addition to the ester C=O peak at 1,739 cm^−1^. Moreover, the ^1^H-NMR spectra of **17a** and **17b** showed a singlet a δ ~ 3.10 (D_2_O exchangeable) corresponding for the NH_2_ protons. Compound **17b** showed a triplet at δ 1.20 for the ester CH_3_ group and a quartet at δ 4.13 ppm for the ester CH_2_ group. The mass spectra of **17a,b** displayed molecular ion peaks at *m/z* 415 and *m/z* 462 corresponding to their respective [M^+^]. A base peak at *m/z* 178 due to fragmentation of [C(=O)-C(N=NPh)=C(NH_2_)-C(CN)=C(NH_2_)]^+^ (*m/z* 239) from [M^+^ + 2] was detected in the mass spectrum of **17a**, while compound **17b** displayed a base peak at *m/z* 133 which corresponds to [CN-C=C-NH-C(=O)-C=C-NH_2_]^+^. A common fragment ion *m/z* 349 was observed in both mass spectra of **17a** and **17b** due to fragmentation of [CN-C=C-NH_2_]^+^ (*m/z* 66) and of [CO_2_Et-C=C-NH_2_]^+^ (*m/z* 113) from their respective [M^+^]. The peak observed with **17a** at *m/z* 106 is attributed to splitting of the phenylhydrazono moiety [N-NHPh]^+^.

### 2.2. Biology

#### 2.2.1. *In vitro* evaluation of antiproliferative activity of the synthesized compounds

The tumor cell growth inhibition activities of the newly synthesized thiophene systems (22 compounds in total) were assessed *in vitro* [34] on three human tumor cell lines, namely, MCF-7 (breast adenocarcinoma), NCI-H460 (non-small cell lung cancer), and SF-268 (CNS cancer) after a continuous exposure of 48 h. The results were compared to the antiproliferative effects of the reference control doxorubicin [35]. All the compounds were dissolved in DMSO at 1 mg/mL immediately before use and diluted just before addition to the cell culture.

The data (Table 1) represents means ± SEM of three independent experiments performed in duplicate. The results indicated that most compounds demonstrated substantial growth inhibitory effects against the human tumor cells at the concentrations tested. The antiproliferative activity of the test compounds against each of the title tumor cell lines may be arranged in a descending order due to measured concentration required to inhibit tumor cell proliferation by 50% (GI_50_ µ/M).

**Table 1:** Antiproliferative activity GI_50_ (µM) of the synthesized compounds.

Compound	GI_50_ (µM) ^a^		
	MCF-7	NCI-H460	SF-268
**2**	30.0 ± 0.6	19.3 ± 1.4	26.3 ± 1.5
**3**	44.6 ± 12.6	32.6 ± 8.6	60.4 ± 14.8
**4**	10.8 ± 0.6	16.5 ± 0.8	16.7 ± 1.6
**5**	75.7 ± 17.5	40.2 ± 12.8	52.0 ± 9.0
**6**	37.4 ± 10.2	22.1 ± 0.8	14.9 ± 6.8
**7a**	2.5 ± 0.5	10.4 ± 0.6	8.0 ± 0.4
**7b**	74.9 ± 0.9	40.6 ± 1.8	58.8 ± 0.8
**9a**	38.0 ± 1.8	40.0 ± 0.8	22.5 ± 1.1
**9b**	20.0 ± 0.2	30.6 ± 1.4	38.4 ± 0.6
**10a**	16.7 ± 1.6	10.8 ± 0.6	16.5 ± 0.8
**10b**	50.1 ± 0.7	23.2 ± 4.8	18.4 ± 1.8
**10c**	39.0 ± 1.8	46.0 ± 0.8	22.5 ± 1.1
**10d**	22.0 ± 0.2	30.6 ± 1.4	38.4 ± 0.6
**11a**	66.6 ± 12.2	12.0 ± 6.2	24.8 ± 3.2
**11b**	22.0 ± 0.4	26.3 ± 0.8	39.0 ± 0.8
**12**	10.9 ± 0.2	146.1 ± 0.6	22.3 ± 0.5
**13**	42.6 ± 12.2	32.6 ± 8.6	64.4 ± 14.8
**14a**	20.0 ± 0.2	32.6 ± 1.4	36.4 ± 0.6
**14b**	11.8 ± 0.6	14.5 ± 0.8	16.7 ± 1.6
**15**	36.4 ± 10.2	20.1 ± 0.8	18.9 ± 6.8
**17a**	2.0 ± 0.4	8.3 ± 0.8	4.0 ± 0.8
**17b**	68.6 ± 12.2	12.0 ± 6.2	24.8 ± 3.2
*Doxorubicin	0.0428 ± 0.0082	0.0940 ± 0.0087	0.0940 ± 0.0070

^a ^Drug concentration required to inhibit tumor cell proliferation by 50% after continuous exposure of 48 h; data are expressed as means ±SEM of three independent experiments performed in duplicates; ^*^Doxorubicin was used as positive control.

The sequence "tumor cell ", [test compound number] and (GI_50_ µ/M) followed the sequence:

**"MCF-7" [Doxorubicin]** (0.0428 ± 0.0082), **[17a]** (2.0 ± 0.4), **[7a]** (2.5 ± 0.5), **[4]** (10.8 ± 0.6), **[12]** (10.9 ± 0.2), **[14b]** (11.8 ± 0.6), **[10a]** (16.7 ± 1.6), **[9b, 14a]** (20.0 ± 0.2), **[10d]** (22.0 ± 0.2), **[11b]** (22.0 ± 0.4), **[2]** (30.0 ± 0.6), **[15]** (36.4 ± 10.2), **[6]** (37.4 ± 10.2), **[9a]** (38.0 ± 1.8), **[10c]** (39.0 ± 1.8), **[13]** (42.6 ± 12.2), **[3]** (44.6 ± 12.6), **[10b]** (50.1 ± 0.7), **[11a]** (66.6 ± 12.2), **[17b]** (68.6 ± 12.2), **[7b]** (74.9 ± 0.9), **[5]** (75.7 ± 17.5).

**"NCI-H460" [Doxorubicin]** (0.0940 ± 0.0087), **[17a]** (8.3 ± 0.8), **[7a]** (10.4 ± 0.6), **[10a]** (10.8 ± 0.6), **[11a, 17b]** (12.0 ± 6.2), **[14b]** (14.5 ± 0.8), **[4]** (16.5 ± 0.8), **[2]** (19.3 ± 1.4), **[15]** (20.1 ± 0.8), **[6]** (22.1 ± 0.8), **[10b]** (23.2 ± 4.8), **[11b]** (26.3 ± 0.8), **[10d, 9b]** (30.6 ± 1.4), **[14a]** (32.6 ± 1.4), **[13, 3]** (32.6 ± 8.6), **[9a]** (40.0 ± 0.8), **[5]** (40.2 ± 12.8), **[7b]** (40.6 ± 1.8), **[10c]** (46.0 ± 0.8).

**"SF–268" [Doxorubicin]** (0.0940 ± 0.0070), **[17a]** (4.0 ± 0.8), **[7a]** (8.0 ± 0.4), **[6]** (14.9 ± 6.8), **[10a]** (16.5 ± 0.8), **[4, 14b]** (16.7 ± 1.6), **[10b]** (18.4 ± 1.8), **[15]** (18.9 ± 6.8), **[12]** (22.3 ± 0.5), **[9a, 10c]** (22.5 ± 1.1), **[11a, 17b]** (24.8 ± 3.2), **[2]** (26.3 ± 1.5), **[14a]** (36.4 ± 0.6), **[9b, 10d]** (38.4 ± 0.6), **[11b]** (39.0 ± 0.8), **[5]** (52.0 ± 9.0), **[7b]** (58.8 ± 0.8), **[3]** (60.4 ± 14.8), **[13]** (64.4 ± 14.8).

In general, compounds **17a**, **7a**, **14b**, **4**, and **10a** showed significant activity on the three tumor cell lines tested. The inhibitory effect of the other systems on tumor cell growth varied, according to the tested tumor cell, from high to medium or marginal effects. Some compounds had no impact on a specific tumor cell proliferation, while exhibited some specificity to the other. Thus compound **11a** revealed GI_50_ ~ 66.6 µ/M towards MCF-7 tumor cell versus GI_50_ ~ 12.0 µ/M for NCI-H460. Similarly, compound **12** had no effect on NCI-H460 tumor cell proliferation (GI_50_ ~ 146 µ/M) while it showed high selectivity towards breast derived cells MCF-7 (GI_50_ ~ 10.9 µ/M).

It is of interest that the pyrazole derivative **7a**, comprising one phenyl substituent, showed significant growth inhibition activity on the three tumor cell lines, compared to its counterpart **7b** with two phenyl functions. Also, comparing the 5-cyano pyridone derivative **17a** and its 5-ethoxy-carbonyl counterpart **17b**, it is obvious that the former has the highest inhibitory activity towards adenocarcinoma (MCF-7), while **17b** showed the lowest effect on the same tumor cell line.

## 3. Experimental

### 3.1. General

All melting points were determined on an Electrothermal digital melting point apparatus and are uncorrected. IR spectra (KBr discs) were recorded on a FTIR plus 460 or Pye Unicam SP-1000 spectrophotometer. ^1^H-NMR spectra were recorded with Varian Gemini -200 (200 MHz) and Jeol AS 500 MHz instruments in DMSO-d6 as solvent using TMS as internal standard and chemical shifts are expressed as δ ppm. The mass spectra were recorded with Hewlett Packard 5988 A GC/MS system and GCMS-QP 1000 Ex Shimadzu instruments. Analytical data were obtained from the Micro-analytical Data Unit at Cairo University and were performed on Vario EL III Elemental CHNS analyzer.

### 3.2. Chemistry

#### 3.2.1. 2-Cyano-*N*-(3-cyano-4,5,6,7-tetrahydrobenzo[*b*]thiophen-2-yl)-acetamide (**2**)

To a solution of 2-amino-3-cyano-4,5,6,7-tetrahydrobenzo[*b*]thiophene (**1**) (1.78 g, 0.01 mol) in dimethylformamide (30 mL), ethyl cyanoacetate (1.13 g, 0.01 mol) was added. The reaction mixture was heated under reflux for 5 h. The solid product formed upon pouring onto ice/water mixture was collected by filtration and crystallized from 1,4-dioxane. Pale yellow crystals, m.p. 129-130 °C, yield: 1.79 g (73%); Anal. For C_12_H_11_N_3_OS (245.30), (% Calcd./Found): 58.76/59.01 (C), 4.52/4.90 (H), 17.13/16.89 (N), 13.07/13.45 (S); IR (*υ*, cm^−1^): 3,431-3,217 (NH), 3,090–3,010 (CH aromatic); 2,938–2,837 (CH_2_), 2,262, 2,196 (2CN), 1,696 (C=O), 1,581, 1,434 (C=C); ^1^H-NMR (δ, ppm): 1.70–2.60 (m, 8H, cyclohexene 4CH_2_), 4.11 (s, 2H, CH_2_), 6.94 (s, 1H, NH); MS *m/z* (%): 245 [M^+^] (38.70), 178{[M^+^ + 1] − [C_3_H_2_NO]^+^}(75.80), 150 {[M^+^] − [C_4_H_3_N_2_O]^+^} (100.00), 116 (11.80), 68 [C_3_H_2_NO]^+^ (13.60).

#### 3.2.2. 4-Amino-2-hydroxy-5,6,7,8-tetrahydrobenzo[4,5]thieno[2,3-*b*] pyridine-3-carbonitrile (**3**)

A solution of **2** (2.45 g, 0.01 mol) in 1,4-dioxane (20 mL) containing triethylamine (2 mL) was heated under reflux for 5 h. The solid product formed upon pouring onto ice/water was collected by filtration, and crystallized from 1,4-dioxane. Brown crystals, m.p. 90–94 °C, yield: 1.52 g (65%); Anal. For C_12_H_11_N_3_OS (245.30), (% Calcd./Found): 58.76/58.77 (C), 4.52/4.85 (H), 17.13/17.00 (N), 13.07/13.30 (S); IR (*υ*, cm^−1^): 3,432 (OH, enol form); 3,333–3,218 (NH, NH_2_), 3,100 (CH aromatic), 2,934-2,839 (CH_2_), 2,197 (CN), 1,621 (C=O, keto form), 1,574, 1,438 (C=C); ^1^H-NMR (δ, ppm):1.75–2.55 (m, 8H, cyclohexene 4CH_2_), 3.61 (s, 2H, NH_2_), 6.97 (s, 1H, ring NH) ); MS *m/z* (%): 245 [M^+^] (100.00), 192 (90.35), 164 (35.09), 136 (93.72), 68 [C_3_H_2_NO]^+^ (63.27).

#### 3.2.3. Synthesis of the amide derivatives **4** and **5**.

To a solution of **2** (2.45 g, 0.01 mol) in 1,4-dioxane (25 mL) containing piperidine (1.00 mL) either salicylaldehyde (1.22 g, 0.01 mol) or benzaldehyde (1.06 g, 0.01 mol) was added. The reaction mixture, in each case, was heated under reflux for 5 h. The solid products formed upon pouring onto ice-water mixture containing few drops of hydrochloric acid was collected by filtration and crystallized from 1,4-dioxane. 

*N-(3-cyano-4,5,6,7-tetrahydrobenzo[b]thiophen-2-yl)-2-oxo-2H-chromen-3-il-carboxamide* (**4**). Orange crystals, m.p. 271-273 C, yield: 2.63 g (75%); Anal. For C_19_H_14_N_2_O_3_S (350.39), (% Calcd./Found): 65.13/64.76 (C), 4.03/4.32 (H), 7.99/8.25 (N), 9.15/9.27 (S); IR (*υ*, cm^−1^): 3,324 (NH), 3,105–3,027 (CH aromatic), 2,928–2,857 (CH_2_), 2,210 (CN), 1,715, 1,678 (2C=O), 1,569, 1,448 (C=C); ^1^H-NMR (δ, ppm): 1.84–2.72 (m, 8H, cyclohexene 4CH_2_), 6.94 (s, 1H, coumarin C4-H), 7.03–7.53 (m, 4H, C_6_H_4_), 8.50 (s, 1H, NH); MS *m/z* (%): 351 [M^+^ + 1] (1.50), 350 [M^+^] (8.40), 349 [M^+^ − 1] (18.00), 316 (34.90) , 172 {[M^+^ − 1] − [C_9_H_9_N_2_S]^+^} (100.00), 89 (67.80).

*2-Cyano-N-(3-cyano-4,5,6,7-tetrahydrobenzo[b]thiophen-2-yl)-3-phenylacrylamide* (**5**). Pale yellow crystals, m.p. 88-92 °C, yield: 2.50 g (75%); Anal. for C_19_H_15_N_3_OS (333.41), (% Calcd./Found): 68.45/ 68.11 (C), 4.53/4.80 (H), 12.60/12.46 (N), 9.62/9.98 (S); IR (υ, cm^–1^): 3,348–3,210 (NH), 3,059 (CH aromatic), 2,933–,2855 (CH aliphatic, CH_2_), 2,244, 2,214 (2CN), 1,620 (C=O), 1,571, 1,447 (C=C); ^1^H-NMR (δ, ppm): 1.77–2.66 (m, 8H, cyclohexene 4CH_2_), 6.87 (s, 1H, NH), 7.00–7.97 (m, 5H, C_6_H_5_), 8.56 (s, 1H, benzylidene CH); MS *m/z* (%): 333 [M^+^] (12.50), 266 {[M^+^ − 1] − [C_3_NO]^+^} (100.00), 238 (77.40), 66 [C_3_NO]^+^ (1.90), 77 [C_6_H_5_]^+^ (33.40).

#### 3.2.4. 2-Cyano-*N*-(3-cyano-4,5,6,7-tetrahydrobenzo[*b*]thiophen-2-yl)-3-phenylbut-2E-enamide (**6**).

To a mixture of equimolar amounts of **2** (2.45 g, 0.01 mol) and acetophenone (1.20 g, 0.01 mol), ammonium acetate (0.50 g) was added and the reaction mixture was heated in an oil bath 140 ^o^C for 45 min. The reaction mixture was then boiled in ethanol (60 mL) for few minutes, poured onto ice/water mixture and the formed product was crystallized from ethanol. Pale brown crystals, m.p. 118–120 °C, yield: 2.08 g (60%); Anal. For C_20_H_17_N_3_OS (347.44), (% Calcd./Found): 69.14/69.40 (C), 4.93/5.12 (H), 12.09/12.07 (N), 9.23/9.51 (S); IR (*υ*, cm^−1^): 3,428–3,220 (NH), 3,082-3,000 (CH aromatic), 2,935–2,840 (CH aliphatic), 2,216, 2,198 (2CN), 1,692 (C=O), 1,576, 1,438 (C=C); ^1^H- NMR (δ, ppm): 1.66-2.35 (m, 8H, cyclohexene 4CH_2_), 1.87 (s, 3H, CH_3_), 6.90 (s, 5H, C_6_H_5_), 11.50 (s, 1H, NH); MS *m/z* (%): 346 [M^+^ − 1] (1.92), 178 {[M^+^ + 1] − [C_11_H_8_NO]^+^} (55.94), 150 {[M^+^] − [C_12_H_9_N_2_O]^+^} (100.00), 116 (8.76), 77 [C_6_H_5_]^+^ (14.92). 

#### 3.2.5. Synthesis of pyrazole carboxamide derivatives **7a,b**

To a solution of compound **4** (3.33 g, 0.01 mol) in 1,4-dioxane (25 mL) and dimethylformamide (10 mL), either hydrazine hydrate (0.50 g, 0.01 mol), or phenyl hydrazine (1.08 g, 0.01 mol) was added. The reaction mixture, in each case, was heated under reflux for 5 h. The solid products formed, in each case, upon pouring onto ice/water mixture containing few drops of hydrochloric acid were collected by filtration, and crystallized from 1,4-dioxane/dimethylformamide mixture. 

*3-Amino-N-(3-cyano-4,5,6,7-tetrahydrobenzo[b]thiophen-2-yl)-5-phenyl-1H-pyrazol-4-yl-carbox-amide* (**7a**). Pale brown crystals, m.p. 231-235 °C, yield: 2.54 g (70%); Anal. For C_19_H_17_N_5_OS (363.44), (% Calcd./Found): 62.79/63.18 (C), 4.71/5.05 (H), 19.27/19.33 (N), 8.82/9.20 (S); IR (*υ*, cm^−1^): 3,445–3,220 (2NH, NH_2_), 3,050 (CH aromatic), 2,929–2,848 (CH aliphatic, CH_2_), 2,209 (CN), 1,622 (C=O), 1,550, 1,439 (C=C); ^1^H-NMR (δ, ppm): 1.71–2.70 (m, 8H, cyclohexene 4CH_2_), 3.90 (s, 2H, NH_2_), 6.81 (s, 1H, NH), 7.17–7.86 (m, 5H, C_6_H_5_), 8.68 (s, 1H, NH). 

*3-Amino-N-(3-cyano-4,5,6,7-tetrahydrobenzo[b]thiophen-2-yl)-1,5-diphenyl-1H-pyrazol-4-yl-carboxamide* (**7b**). Pale orange crystals, m.p. 128-130 °C, yield: 3.34 g (76%); Anal. For C_25_H_21_N_5_OS (439.53), (% Calcd./Found): 68.32/68.00 (C), 4.82/4.53 (H), 15.93/15.63 (N), 7.30/7.62 (S); IR (*υ*, cm^−1^): 3,452–3,225 (NH, NH_2_), 3,056–3,028 (CH aromatic), 2,930-2,851 CH_2_), 2,209 (CN), 1,621 (C=O), 1,597, 1,441 (C=C); ^1^H-NMR (δ, ppm): 1.71–2.69 (m, 8H, cyclohexene 4CH_2_), 3.90 (s, 2H, NH_2_), 7.02-7.88 (m, 10H, 2C_6_H_5_), 10.29 (s, 1H, NH); MS *m/z* (%): 440 [M^+^ + 1] (9.35), 439 [M^+^] (12.03), 423 (83.97), 219 (100.00), 150 (42.43).

#### 3.2.6. Synthesis of 3-cyano-4,5,6,7-tetrahydrobenzo[b]thiophen-2-yl-functionalized pyridone derivatives **9a,b**.

Method (A): To a solution of **4** (3.33 g, 0.01 mol) in 1,4-dioxane (25 mL) and dimethylformamide (5 mL) containing triethylamine (1.00 mL), either malononitrile (0.66 g, 0.01 mol) or ethyl cyanoacetate (1.13 g, 0.01 mol) was added. The reaction mixture, in each case, was heated under reflux for 5 h, then cooled and neutralized by pouring onto ice/water mixture containing few drops of hydrochloric acid. The solid products formed, in each case, was filtered off and crystallized from 1,4-dioxane/dimethylformamide mixture. 

Method (B): Equimolar amounts of **2** (2.45 g, 0.01 mol) and either benzylidene malononitrile (1.54 g, 0.01 mol) or benzylidene ethyl cyanoacetate (2.01g, 0.01 mol) in 1,4-dioxane (25 mL) and dimethylformamide (5 mL) containing triethylamine (1.00 mL) were heated under reflux for 5 h. The reaction mixture in each case was treated in a similar manner as in method A.

*6-Amino-1-(3-cyano-4,5,6,7-tetrahydrobenzo[b]thiophen-2-yl)-2-oxo-4-phenylpyridine-3,5-dicarbonitrile* (**9a**). Pale yellow crystals, m.p. > 300 °C, yield: (2.98 g 75%, method A; 3.18 g 80% method B); Anal. For C_22_H_15_N_5_OS (397.45), (% Calcd./Found): 66.48/66.88 (C), 3.80/3.90 (H), 17.62/17.38 (N) , 8.07/8.30 (S); IR (*ν*, cm^−1^): 3,307, 3,218 (NH_2_), 3,061 (CH aromatic); 2,938-2,862 (CH_2_), 2,253, 2,223, 2,209 (3CN), 1,632 (C=O), 1,572, 1,484 (C=C); ^1^H-NMR (δ, ppm): 1.88-2.55 (m, 8H, cyclohexene 4CH_2_), 3.61 (s, 2H, NH_2_), 7.54–7.63 (m, 5H, C_6_H_5_); MS *m/z* (%): 399 [M^+^ + 2] (13.30), 398 [M^+^ + 1) (21.30), 397 [M^+^] (100.00), 199 (14.90), 150 (12.80), 119 (11.70).

*Ethyl 2-amino-5-cyano-1-(3-cyano-4,5,6,7-tetrahydrobenzo[b]thiophen-2-yl)-1,6-dihydro-6-oxo-4-phenylpyridine-3-carboxylate* (**9b**). Pale yellow crystals, m.p. > 300 °C, yield: (3.24 g 73%, method A; 3.82 g 86 %, method B); Anal. For C_24_H_20_N_4_O_3_S (444.51), (% Calcd./Found): 64.85/65.00 (C), 4.54/4.92(H), 12.60/12.21 (N), 7.21/7.43 (S); IR (*υ*, cm^−1^): 3,326-3,214 (NH_2_), 3,060 (CH aromatic), 2,933-2,855 (CH_2_, CH_3_), 2,218, 2,204 (CN), 1,695, 1,634 (2C=O), 1,573, 1,446 (C=C); ^1^H-NMR (δ, ppm): 1.21(t, *J* = 8.00 Hz, 3H, CH_3_), 1.82-2.73 (m, 8H, cyclohexene 4CH_2_), 3.41 (s, 2H, NH_2_), 4.30 (q, *J* = 8.00 Hz, 2H, CH_2_), 6.98–8.02 (m, 5H, C_6_H_5_); MS *m/z* (%): 444 [M^+^] (43.15), 407 (66.02), 242 (80.91), 198 (79.73), 73 (72.50), 61 (100.00).

#### 3.2.7. Synthesis of 3-cyano-4,5,6,7-tetrahydrobenzo[*b*]-thiophen-2-yl-functionalized 2-pyridone derivatives **10a-d**.

The procedure adopted for the synthesis of **9a,b** (Method A) was followed using **2** (2.45 g, 0.01 mol) and either malononitrile (0.66 g, 0.01 mol), ethyl cyanoacetate (1.13 g, 0.01 mol), acetyl acetone (1.00 g, 0.01 mol) or ethyl acetoacetate (1.33 g, 0.01 mol). The solid products formed, in each case, upon treating of the reaction mixtures was crystallized from ethanol/dimethylformamide mixture.

*4,6-Diamino-1-(3-cyano-4,5,6,7-tetrahydrobenzo[b]thiophen-2-yl)-1,2-dihydro-2-pyridone-3-carbonitrile* (**10a**). Brown crystals, m.p. 215-218 °C, yield: 2.33 g (75%); Anal. For C_15_H_13_N_5_OS (311.36), (% Calcd./Found): 57.86/58.04 (C), 4.21/4.50 (H), 22.49/ 22.10 (N), 10.30/10.62 (S); IR (*υ*, cm^−1^): 3,430–3,217 (2NH_2_), 3,010 (CH aromatic), 2,936–2,836 (CH_2_), 2,250, 2,195 (2CN), 1,620 (C=O), 1,574, 1,433 (C=C); ^1^H-NMR (δ, ppm): 1.70–2.69 (m, 8H, cyclohexene 4CH_2_), 2.91, 2.98 (2s, 4H, 2NH_2_), 7.89 (s, 1H, pyridine C5-H); MS *m/z* (%): 311 [M^+^] (49.14), 296 {[M^+^ + 1] − (NH_2_)} (55.49), 282 {[M^+^ + 3] − (2NH_2_)} (46.55), 136 (77.59), 60 (100.00).

*4-Amino-1-(3-cyano-4,5,6,7-tetrahydrobenzo[b]thiophen-2-yl)-6-hydroxy-1,2-dihydro-2-pyridone-3-carbonitrile* (**10b**). Pale yellow crystals, m.p. 277–280 °C, yield: 2.00 g (64%); Anal. For C_15_H_12_N_4_O_2_S (312.34), (% Calcd./Found): 57.68/57.83 (C), 3.87/4.22 (H), 17.94/17.54 (N), 10.27/10.50 (S); IR (*υ*, cm^−1^): 3,431 (OH), 3,334–3,224 (NH_2_), 3,093 (CH aromatic), 2,935–2,840, (CH_2_), 2,219, 2,199 (2CN), 1,694 (C=O), 1,581, 1,438 (C=C); ^1^H-NMR (δ, ppm): 1.79-2.64 (m, 8H, cyclohexene 4CH_2_), 4.11 (s, 2H, NH_2_), 6.92 (s, 1H, pyridine C5-H), 11.89 (s, 1H, OH); MS *m/z* (%): 312 [M^+^] (19.44), 311 [M^+^ − 1] (100.00), 296 {[M^+^] − (NH_2_)} (49.67), 285 {[M^+^ − 1] − [CN]^+^} (30.17), 219 {[M^+^ + 1] − [C_4_H_2_N_2_O]^+^} (73.86), 164 {[M^+^ + 3] − [C_6_H_5_N_3_O_2_]^+^} (66.06).

*1-(3-Cyano-4,5,6,7-tetrahydrobenzo[b]thiophen-2-yl)-4,6-dimethyl-1,2-dihydro-2-pyridone-3-carbonitrile* (**10c**). Brown crystals, m.p. 130–135 °C, yield: 2.25 g (73%); Anal. For C_17_H_15_N_3_OS (309.39), (% Calcd./Found): 66.00/66.20 (C), 4.89/5.19 (H), 13.58/13.48 (N), 10.36/ 10.51 (S); IR (*υ*, cm^−1^): 3,074 (CH aromatic), 2,933, 2,851 (CH_2_, CH_3_), 2,215, 2,230 (2CN), 1,667 (C=O), 1,572, 1,439 (C=C); ^1^H- NMR (δ, ppm): 1.77-2.60 (m, 8H, cyclohexene 4CH_2_), 2.11, 2.36 (2s, 6H, 2CH_3_), 6.51 (s, 1H, pyridine C5-H); MS *m/z* (%): 311 [M^+^ + 2] (7.58), 310 [M^+^ + 1] (20.59), 309 [M^+^] (100.00), 281 {[M^+^ − 2] − [CN]^+^} (74.34), 162 [C_9_H_8_NS]^+^ (28.01), 147 [C_8_H_7_N_2_O]^+^ (5.08), 131 {[M^+^] − [C_9_H_10_N_2_S]} (38.92).

*1-(3-Cyano-4,5,6,7-tetrahydrobenzo[b]thiophen-2-yl)-6-hydroxy-4-methyl-1,2-dihydro-2-pyridone-3-carbonitrile* (**10d**). Pale brown crystals, m.p. 251–255 °C, yield: 2.02 g (65%); Anal. For C_16_H_13_N_3_O_2_S (311.35), (% Calcd./Found): 61.72/61.52 (C), 4.21/4.30 (H), 13.50/13.87 (N), 10.30/10.64 (S); IR (*υ*, cm^−1^): 3,445-3,225 (OH), 2,931-2,850 (CH_2_, CH_3_), 2,220, 2,209 (CN), 1,668 (C=O), 1,574, 1,440 (C=C); ^1^H-NMR (δ, ppm): 1.82-2.94 (m, 8H, cyclohexene 4CH_2_), 2.55 (s, 3H, CH_3_), 6.88 (s, 1H, pyridine C5-H), 12.10 (s, 1H, OH); MS *m/z* (%): 313 [M^+^ + 2] (1.08), 312 [M^+^ + 1] (0.75), 311 [M^+^] (0.80), 219 {[M^+^ + 1] − [C_5_H_3_NO]^+^} (100.00), 163 [C_9_H_9_NS]^+^ (13.47), 150 [C_7_H_6_N_2_O_2_]^+^ (48.18).

#### 3.2.8. Synthesis of (3-cyano-4,5,6,7-tetrahydrobenzo[*b*]thiophen-2-yl)- functionalized thiophene- derivatives **11a,b** and the thiazole derivative **12**.

To a solution of compound **2** (2.45 g, 0.01 mol) in 1,4-dioxane (25 mL) containing triethylamine (1.00 mL), either malononitrile (0.66 g, 0.01 mol), ethyl cyanoacetate (1.13 g, 0.01 mol) or phenyl isothiocyanate (1.35 g, 0.01 mol) was added followed by the addition of an equimolar amount of elemental sulfur (0.32 g, 0.01 mol). The reaction mixture was heated under reflux for 5 h, then cooled and neutralized by pouring onto ice/water mixture containing few drops of hydrochloric acid. The solid product formed in each case was collected by filtration and crystallized from dimethylformamide. 

*3,5-Diamino-4-cyano-N-(3-cyano-4,5,6,7-tetrahydrobenzo[b]thiophen-2-yl)thiophene-2-carboxamide* (**11a**). Brown crystals, m.p. > 300 °C, yield: 2.06 g (60%); Anal. For C_15_H_13_N_5_OS_2_ (343.43), (% Calcd./Found): 52.46/52.80 (C), 3.82/3.73 (H), 20.39/20.16 (N), 18.67/18.41 (S); IR (*υ*, cm^−1^): 3431–3216 (NH, 2NH_2_), 2,933-2,841 (CH_2_), 2,202, 2,195 (2CN), 1,632 (C=O), 1,572, 1,407 (C=C); ^1^H-NMR (δ, ppm): 1.67–2.49 (m, 8H, cyclohexene 4CH_2_), 3.34, 3.38 (2s, 4H, 2NH_2_), 6.90 (s, 1H, NH); MS *m/z* (%): 346 [M^+^ + 3] (0.88), 178 [C_9_H_10_N_2_S] (39.83), 163 (1.58), 150 [C_8_H_8_NS]^+^ (100.00), 60 (10.38). 

*Ethyl 5-(3-cyano-4,5,6,7-tetrahydrobenzo[b]thiophen-2-yl-carbamoyl)-2,4-diaminothiophene-3-carboxylate* (**11b**). Brown crystals, m.p. 163-166 °C, yield: 2.54 g (65%); Anal. For C_17_H_18_N_4_O_3_S_2_ (390.47), (% Calcd./Found): 52.29/52.00 (C), 4.65/4.46 (H), 14.35 / 14.00 (N), 16.42/16.81 (S); IR (υ, cm^−1^): 3,324–3,221 (NH, 2NH_2_), 2,929, 2,848 (CH_2_, CH_3_), 2,205 (CN), 1,696, 1,625 (2C=O), 1,571, 1,438 (C=C); ^1^H-NMR (δ, ppm): 1.12 (t, *J* = 7.00 Hz, 3H, CH_3_), 1.72-2.50 (m, 8H, cyclohexene 4CH_2_), 3.31, 3.35 (2s, 2H each, 2NH_2_), 3,49 (q, *J* = 7.00 Hz, 2H, CH_2_), 6.90 (s, 1H, NH); MS *m/z* (%): 392 [M^+^ + 2] (36.61), 391 [M^+^ + 1] (8.09), 362 {[M^+^] − [CH_2_N]^+^} (100.00), 178 [C_9_H_10_N_2_S] (14.57), 150 [C_8_H_8_NS]^+^ (28.83), 127 (77.20).

*4-Amino-N-(3-cyano-4,5,6,7-tetrahydrobenzo[b]thiophen-2-yl)-2,3-dihydro-3-phenyl-2-thioxothiazol-5-yl-carboxamide* (**12**). Dark brown crystals, m.p. 103-105 °C, yield: 3.01 g (73%); Anal. For C_19_H_16_N_4_OS_3_ (412.55), (% Calcd./Found): 55.32/55.51 (C), 3.91/3.85 (H), 13.58/13.34 (N), 23.32/23.15 (S); IR (*υ*, cm^−1^): 3,319 (NH, NH_2_), 2,927–2,847 (CH_2_), 2,201 (CN), 1,632 (C=O), 1,539, 1,437 (C=C), 1,327, 1,282 (C=S); ^1^H-NMR (δ, ppm): 1.72-2.85 (m, 8H, cyclohexene 4CH_2_), 3.41 (s, 2H, NH_2_), 7.10–7.59 (m, 5H, C_6_H_5_), 7.92 (s, 1H, NH); MS *m/z* (%): 412 [M^+^] (0.23), 178 [C_9_H_10_N_2_S] (62.74), 150 [C_8_H_8_NS]^+^ (74.81), 126 (84.47), 93 [Ph-NH_2_] (100.00), 77 [C_6_H_5_]^+^ (29.64). 

#### 3.2.9. *2-(4-Amino-2,3-dihydro-6-oxo-3-phenyl-2-thioxopyrimidin-1(6H)-yl)-4,5,6,7-tetrahydrobenzo[b]thiophene-3-carbonitrile* (**13**).

Equimolar amounts of **2** (2.45 g, 0.01 mol) and phenyl isothiocyanate (1.35 g, 0.01 mol) in 1,4-dioxane (20 mL) containing triethylamine (1.0 mL) were heated under reflux for 5h. After cooling, the reaction mixture was acidified by hydrochloric acid and the crude product was precipitated, collected by filtration and crystallized from dimethylformamide. Pale yellow crystals, m.p. > 300 °C, yield: 3.04 g (80%); Anal. For C_19_H_16_N_4_OS_2_ (380.49), (% Calcd./Found): 59.98/59.60 (C), 4.24/4.27 (H), 14.73/ 14.33 (N), 16.85/17.20 (S); IR (*υ*, cm^−1^): 3,442–3,242 (NH_2_), 3,056 (CH aromatic), 2,931, 2,837 (CH_2_), 2,206 (CN), 1,607 (C=O), 1,561, 1,498 (C=C); 1,373, 1,280 (C=S); ^1^H-NMR (δ, ppm): 1.88–2.70 (m, 8H, cyclohexene 4CH_2_), 3.83 (s, 2H, NH_2_), 6.92 (s, 1H, pyrimidine C3-H), 7.12-7.62 (m, 5H, C_6_H_5_); MS *m/z* (%): 382 [M^+^ + 2] (8.20), 313 {[M^+^ + 2] − [C_3_H_3_NO]^+^} (100.00), 255 {[M^+^ + 1] − [C_4_H_2_N_2_OS]^+^} (37.00), 226 (11.00), 147 (11.60), 126 (19.20).

#### 3.2.10. Synthesis of the 3-cyano-4,5,6,7-tetrahydrobenzo[*b*]thiophen-2-yl-functionalized thiophene derivatives **14a,b** and the thiazole **15** derivative.

Equimolar amounts of **2** (2.45 g, 0.01 mol) and phenyl isothiocyanate (1.35 g, 0.01 mol) in dimethylformamide (20 mL) and potassium hydroxide were stirred overnight, then added ethyl chloroacetate (1.22 g, 0.01 mol), phenacyl bromide (1.99 g, 0.01 mol), or chloroacetone (0.92 g, 0.01 mol) while stirring overnight. The solid products formed upon pouring onto ice/water mixture containing few drops of hydrochloric acid were collected by filtration and crystallized from 1,4-dioxane. 

*Ethyl 3-amino-4-(3-cyano-4,5,6,7-tetrahydrobenzo[b]thiophen-2-yl-carbamoyl)-5-(phenylamino)-thio-phene-2-carboxylate* (**14a**). Dark green crystals, m.p. 102–105 °C, yield: 4.57 g (98%); Anal. For C_23_H_22_N_4_O_3_S_2_ (466.57), (% Calcd./Found): 59.21/58.90 (C), 4.75/4.68 (H), 12.01/11.97 (N), 13.74/ 14.00 (S); IR (*υ*, cm^−1^): 3,368–3,186 (2NH, NH_2_), 3,061 (CH aromatic), 2,931–2,857 (CH_2_, CH_3_), 2,208 (CN), 1,735, 1,655 (2C=O), 1,570, 1,497 (C=C); ^1^H-NMR (δ, ppm): 1.21 (t, *J* = 8.00 Hz, 3H, CH_3_), 1.76–2.91 (m, 8H, cyclohexene 4CH_2_), 4.07 (q, *J* = 8.00 Hz, 2H, CH_2_), 4.37 (s, 2H, NH_2_), 6.94 (s, 1H, NH), 7.14-7.96 (m, 5H, C_6_H_5_), 9.99 (s, 1H, NH); MS *m/z* (%): 466 [M^+^] (0.51), 353 (72.06), 243 (70.07), 215 (82.33), 178 [C_9_H_10_N_2_S] (31.66), 150 [M^+^] − [C_15_H_14_N_3_O_3_S]^+^ (40.22), 77 [C_6_H_5_]^+ ^(100.00).

*3-Amino-2-benzoyl-4-[(3-cyano-4,5,6,7-tetrahydrobenzo-[b]thiophene-2-carbonyl)-amino]-5-phenyl-aminothiophene* (**14b**). Dark orange crystals, m.p. 94–96 °C, yield: 4.89 g (98%); Anal. For C_27_H_22_N_4_O_2_S_2_ (498.62), (% Calcd./Found): 65.04/64.92 (C), 4.45/4.17 (H), 11.24/10.95 (N),12.86/ 12.97 (S); IR (*υ*, cm^−1^): 3,318 (2NH, NH_2_); 3,058(CH aromatic); 2,927-2,847 (CH_2_); 2,203 (CN); 1,720, 1,636 (2C=O); 1,549, 1,485 (C=C); ^1^H-NMR (δ, ppm): 1.81–2.55 (m, 8H, cyclohexene 4CH_2_), 4.80 (s, 2H, NH_2_), 7.38-8.03 (m, 10H, 2C_6_H_5_), 8.91, 9.83 (2s, 2H, 2NH); MS *m/z* (%): 498 [M^+^] (3.9), 255 (13.20), 178 [C_9_H_10_N_2_S] (8.70), 150 [M^+^] − [C_19_H_14_N_3_O_2_S]^+^ (5.80), 134 (13.50), 77 [C_6_H_5_]^+ ^(100.00).

*2-Cyano-N-(3-cyano-4,5,6,7-tetrahydrobenzo[b]thiophen-2-yl)-2-(4-methyl-3-phenyl-3H-thiazol-2-yl-idene)-acetamide* (**15**). Dark reddish brown crystals, m.p. 222–226 °C, yield: 4.14 g (99%); Anal. For C_22_H_18_N_4_OS_2_ (418.54), (% Calcd./Found): 63.13/62.81 (C), 4.33/4.36 (H), 13.39/13.54 (N), 15.32/ 15.13 (S); IR (*υ*, cm^−1^): 3,370 (NH), 3,058 (CH aromatic), 2,926-2,845 (CH_3_), 2,203, 2,173 (CN), 1,640 (C=O), 1,542, 1,467 (C=C); ^1^H-NMR (δ, ppm): 1.38 (s, 3H, CH_3_); 1.79-2.50 (m, 8H, cyclohexene 4CH_2_), 6.67 (s, 1H, thiazole C5-H), 7.09-8.00 (m, 5H, C_6_H_5_), 9.82 (s, 1H, NH); MS *m/z* (%): 419 [M^+^ + 1] (2.90), 418 [M^+^] (4.90), 417 [M^+^ − 1] (4.40), 323 (35.40), 241 [M^+^ + 1] − [C_9_H_10_N_2_S] (100.00),178 [C_9_H_10_N_2_S] (22.80), 150 [M^+^] − [C_14_H_10_N_3_OS]^+^ (21.40), 77 [C_6_H_5_]^+^ (93.70). 

#### 3.2.11. *2-Cyano-2-(2-phenylhydrazono)-N-(3-cyano-4,5,6,7-tetrahydrobenzo[b]thiophen-2-yl)-acetamide* (**16**)

To a cold solution (0–5 °C) of **2** (2.45 g, 0.01 mol), in ethanol (20 mL) containing sodium hydroxide (1.00 g) an equimolar amount of diazotized aniline was gradually added while stirring. The solid product formed upon cooling in an ice-bath was collected by filtration, washed with water and crystallized from 1,4-dioxane. Reddish brown crystals, m.p. 128–132 °C, yield: 2.27 g (65%); Anal. For C_18_H_15_N_5_OS (349.41), (% Calcd./Found): 61.87/62.20 (C), 4.33/4.44 (H), 20.04/19.70 (N), 9.18/ 9.39 (S); IR (*υ*, cm^−1^): 3,364–3,228 (2NH), 3,135–3,066 (CH aromatic), 2,929, 2,850 (CH_2_), 2,258, 2,209 (2CN), 1,682 (C=O), 1,601, 1,493 (C=C), 1,545 (=N-NH); ^1^H-NMR (δ, ppm): 1.75-2.50 (m, 8H, cyclohexene 4CH_2_), 6.95-7.86 (m, 5H, C_6_H_5_), 9.14, 10.88 (2s, 1H each, 2NH); MS *m/z* (%): 350 [M^+^ + 1] (4.83), 349 [M^+^] (17.97), 178 {[M^+^ + 1] − [C_9_H_6_N_3_O]^+^} (100.00), 167 {[M^+^ + 5] − [C_9_H_7_N_4_O]^+^} (34.12), 149 {[M^+^ − 1] − [C_10_H_7_N_4_O]^+^} (80.25), 105 (23.09), 77 [C_6_H_5_]^+^ (60.98).

#### 3.2.12. Synthesis of 3-phenylazo-2-pyridone derivatives **17a,b**

To a solution of **16** (3.49 g, 0.01 mol) in ethanol (25 mL) and dimethylformamide (5 mL) containing triethylamine (1.00 mL), either of malononitrile (0.66 g, 0.01 mol) or ethyl cyanoacetate (1.13 g, 0.01 mol) was added. The reaction mixture, in each case, was heated under reflux for 5 h, then cooled and neutralized by pouring onto ice/water mixture containing few drops of hydrochloric acid. The solid products formed, in each case, was filtered off and crystallized from ethanol/ dimethylformamide mixture. 

*5-(2-phenyldiazenyl)-2,4-diamino-1-(3-cyano-4,5,6,7-tetrahydrobenzo[b]thiophen-2-yl)-1,6-dihydro-6-oxopyridine-3-carbonitrile* (**17a**). Dark orange crystals, m.p. 200–205 °C, yield: 3.49 g (84%); Anal. For C_21_H_17_N_7_OS (415.47), (% Calcd./Found): 60.71/61.10 (C), 4.12/4.50 (H), 23.60/23.30 (N), 7.72/ 7.92 (S); IR (*υ*, cm^−1^): 3,356-3,223 (2NH_2_), 3,135–3,063 (CH aromatic), 2,931-2,853 (CH_2_), 2,253, 2,208 (2CN), 1,681 (C=O), 1,600, 1,490 (C=C); ^1^H-NMR (δ, ppm): 1.66–2.60 (m, 8H, cyclohexene 4CH_2_), 3.13, 3.39 (2s, 4H, 2NH_2_), 7.13-7.64 (m, 5H, C_6_H_5_); MS *m/z* (%): 415 [M^+^] (20.58), 349 {[M^+^] − [C_3_H_2_N_2_]^+^} (63.71), 178 {[M^+^ + 2] − [C_12_H_9_N_5_O]^+^} (100.00), 148 [C_6_H_4_N_4_O]^+^ (40.10), 106 [C_6_H_6_N_2_]^+^ (37.06), 66 [C_3_H_2_N_2_]^+^ (6.08). 

*2-(4,6-Ethyl 5-(2-phenyldiazenyl)-2,4-diamino-1-(3-cyano-4,5,6,7-tetrahydrobenzo[b]thiophen-2-yl)-1,6-dihydro-6-oxopyridine-3-carboxylate* (**17b**). Dark orange crystals, m.p. 166–170 °C, yield: 2.77 g (60%); Anal. For C_23_H_22_N_6_O_3_S (462.52), (% Calcd./Found): 59.73/60.00 (C), 4.79/5.03 (H),18.17/ 17.95 (N), 6.93/7.30 (S); IR (*υ*, cm^−1^): 3,355–3,222 (2NH_2_), 3,060 (CH aromatic), 2,931–2,850 (CH_2_, CH_3_), 2,206 (CN), 1,739, 1,677 (2C=O), 1,598, 1,455 (C=C); ^1^H-NMR (δ, ppm): 1.20 (t, *J* = 7.65 Hz, 3H, CH_3_), 1.72-2.54 (m, 8H, cyclohexene 4 CH_2_), 3.07, 3.08 (2s, 2H each, 2NH_2_), 4.13 (q, *J* = 7.65 Hz, 2H, CH_2_), 7.14-7.81 (m, 5H, C_6_H_5_); MS *m/z* (%): 462 [M^+^] (38.76), 349 {[M^+^] − [C_5_H_7_NO_2_]^+^} (44.53), 181 (51.72), 167 (66.72), 133 [C_6_H_3_N_3_O]^+^ (100.00), 113 (35.91).

### 3.3. Biology

*Materials and methods:* Fetal bovine serum (FBS) and L-glutamine, were obtained from Gibco Invitrogen Company (Scotland, UK). RPMI-1640 medium was provided from Cambrex (New Jersey, USA). Dimethyl sulfoxide (DMSO), doxorubicin, penicillin, streptomycin and sulforhodamine B (SRB) were obtained from Sigma Chemical Company. (Saint Louis, MO, USA).

*Samples:* Stock solutions of compounds **2-17b** were prepared in DMSO and kept at −20 ^o^C. Appropriate dilutions of the compounds were freshly prepared just prior the assays. Final concentrations of DMSO did not interfere with the cell growth.

*Cell cultures:* Three human tumor cell lines, MCF-7 (breast adenocarcinoma), NCI-H460 (non-small cell lung cancer), and SF-268 (CNS cancer) were used. MCF-7 was obtained from the European Collection of Cell Cultures (ECACC, Salisbury, UK) and NCI-H460 and SF-268 were kindly provided by the National Cancer Institute (NCI, Cairo, Egypt). They grow as monolayer and routinely maintained in RPMI-1640 medium supplemented with 5% heat inactivated FBS, 2 mM glutamine and antibiotics (penicillin 100 U/mL, streptomycin 100 µg/mL), at 37 ^o^C in a humidified atmosphere containing 5% CO_2_. Exponentially growing cells were obtained by plating 1.5 × 10^5^ cells/mL for MCF-7 and SF-268 and 0.75 × 10^4^ cells/mL for NCI-H460, followed by 24 h of incubation. The effect of the vehicle solvent (DMSO) on the growth of these cell lines was evaluated in all the experiments by exposing untreated control cells to the maximum concentration (0.5%) of DMSO used in each assay.

## 4. Conclusions

In summary, we have developed a convenient synthetic approach to 22 novel highly substituted and polyfunctionalized heterocyclic systems based on 2-cyano-*N*-(3-cyano-4,5,6,7-tetrahydrobenzo[*b*]-thiophen-2-yl)-acetamide. The regioselective attack by different reagents on the cyanoacetamido moiety in the key precursor **2** led to the diversity of the produced systems. All compounds were assessed for their antiproliferative activities on three human cancer cell lines. Most of the systems were found to be promising antiproliferative agents. As a continuation of this work which provides guidance for the development of other new systems based on functionalized 4,5,6,7-tetrahydrobenzo[*b*]-thiophene core, we intended to study other related systems and the results of further pharmacological investigations will be reported in due course.
